# “How I wished to be like Bruce Lee. Eating Disorders and Psychotic Symptoms” A case report

**DOI:** 10.1192/j.eurpsy.2025.1400

**Published:** 2025-08-26

**Authors:** M. Ligero Argudo, I. M. Peso Navarro, C. García Cerdán, C. Munaiz Cossío, R. K. González Bolaños, J. I. de la Iglesia Larrad, N. M. Casado Espada, A. Macia Casas, D. González Parra

**Affiliations:** 1 Psychiatry, Clinical Hospital of Salamanca, Salamanca, Spain

## Abstract

**Introduction:**

Eating disorders (ED) can sometimes present with psychotic symptoms, including delusions and cenesthetic or auditory hallucinations. In a minority of patients, these symptoms may stem from an underlying psychotic disorder, such as schizophrenia, which is more common in males (Bou Khalil R, Hachem D, Richa S. (2011). Eating disorders and schizophrenia in male patients: a review. Eat Weight Disord, 16(3), 150-6). Therefore, early detection and intervention are critical in cases where EDs are accompanied by prodromal or attenuated psychotic symptoms.

**Objectives:**

To present the clinical case of a 14-year-old male with an unspecified eating disorder and high-risk mental state for psychosish

To highlight the importance of early identification and intervention in eating disorders with psychotic features.

**Methods:**

A Pubmed database was used to collect information about psycothic symptoms in EDs, using the terms ‘eating disorder’, ‘pyscosis’ and ‘high risk mental state’.

We present the following clinical case:

A 14-year-old spanish male of Bolivian descent. The patient exhibited a two-year history of food restriction, vigorous exercise, social isolation, and absenteeism from school. Detailed clinical evaluations were performed, documenting his physical, psychological, and behavioral symptoms. The patient’s diagnosis was reevaluated based on emerging psychotic symptoms during hospitalization.

**Results:**

The patient reported intense distress about perceived fat accumulation in his face and trunk, which he believed diminished immediately after exercise. He engaged in excessive physical activity, including jumping rope for at least two hours multiple times a day, and swimming against currents. He also experienced episodes of binge eating followed by purging and compensatory exercise. Social withdrawal, emotional blunting, disorganized biological rhythms, and soliloquies were observed during his admission. Based on these findings, his diagnosis was revised to an unspecified ED with a high-risk mental state for psychosis.

**Conclusions:**

Psychotic symptoms, particularly in restrictive anorexia, can arise during the course of an ED, with malnutrition acting as both a cause and sustaining factor by exacerbating serotonin-dopamine dysregulation (Sarró, S (2018). Those courageous boys: 73 years after the Minnesota starvation experiment. A psychiatrist’s view. Neurosciences and History, 6(1), 28-37). While these symptoms may result from malnutrition, they could also signal the onset of a primary psychotic disorder, with males at higher risk (3.6%). Early detection of attenuated or prodromal psychotic symptoms is essential, and regular reevaluation is recommended, especially during the first months of follow-up, to prevent short-, medium-, and long-term complications.

**Disclosure of Interest:**

None Declared

